# HSP70 Ameliorates Septic Lung Injury via Inhibition of Apoptosis by Interacting with KANK2

**DOI:** 10.3390/biom12030410

**Published:** 2022-03-07

**Authors:** Qing Pei, Wei Ni, Yihang Yuan, Jing Yuan, Xiong Zhang, Min Yao

**Affiliations:** 1 Department of Plastic and Reconstructive Surgery, Shanghai Ninth People’s Hospital, Shanghai Jiao Tong University School of Medicine, Shanghai 200011, China; drqingpei@gmail.com; 2 Wuhan National Laboratory for Optoelectronics, Huazhong University of Science and Technology, Wuhan 430000, China; niwei@hust.edu.cn (W.N.); yuanj@hust.edu.cn (J.Y.); 3 Department of Pharmacology and Chemical Biology, Shanghai Jiao Tong University School of Medicine, Shanghai 200025, China; yyh123@sjtu.edu.cn; 4 Department of Burn, Ruijin Hospital, Shanghai Jiao Tong University School of Medicine, Shanghai 200025, China; 5 Institute of Traumatic Medicine, Shanghai Jiao Tong University School of Medicine, Shanghai 201999, China

**Keywords:** septic lung injury, HSP70, KANK2, apoptosis, inflammation

## Abstract

Acute lung injury is the most common type of organ damage with high incidence and mortality in sepsis, which is a poorly understood syndrome of disordered inflammation. The aims of this study are to explore whether heat shock protein 70 (HSP70), as a molecular chaperone, attenuates the septic lung injury, and to understand the underlying mechanisms. In our study, treatment with HSP70 ameliorated the survival rate, dysfunction of lung, inflammation, and apoptosis in cecal ligation and puncture (CLP)-treated mice as well as in LPS-treated human alveolar epithelial cells. Furthermore, HSP70 interacted with KANK2, leading to reversed cell viability and reduced apoptosis-inducing factor (AIF) and apoptosis. Additionally, knockdown of KANK2 in epithelial cells and deletion of *hsp70.1* gene in CLP mice aggravated apoptosis and tissue damage, suggesting that interaction of KANK2 and HSP70 is critical for protecting lung injury induced by sepsis. HSP70 plays an important role in protection of acute lung injury caused by sepsis through interaction with KANK2 to reduce AIF release and apoptotic cell. HSP70 is a novel potential therapeutic approach for attenuation of septic lung injury.

## 1. Introduction

Sepsis refers to life-threatening organ dysfunction caused by a dysregulated host response to infection. Mostly, it is prone to occur after severe burns, surgery, and infection [1,2]. It has been reported that there are more than 19 million sepsis patients worldwide each year, of which 6 million patients die, and the case fatality rate exceeds 1/4 [3,4]. The primary causes of death in septic multiple organ failure are diffuse inflammation of the lung parenchyma and severe pulmonary dysfunction. Nearly 50% of patients with severe sepsis can develop acute lung injury (ALI) or even acute respiratory distress syndrome (ARDS). The current treatment for sepsis mainly includes timely elimination of local infection foci, strengthening of circulation and respiratory support, early application of antibiotics, etc.; however there is a lack of specific and effective therapies [5,6,7].

ALI is characterized by infiltration of neutrophils, destruction of the barrier between pulmonary capillary and alveolar epithelial cells, and accumulation of protein-rich edema fluid in the alveoli and interstitium, resulting in acute hypoxic respiratory insufficiency. Under the stimulation of infection, inflammatory cells are activated and recruited to the lung interstitial alveolar cavity and release a large amount of proinflammatory mediators such as cytokines, leukotrienes, and peroxides that induce apoptosis and damage lung capillary and alveolar epithelial cells. Apoptosis of numerous endothelial cells caused increased permeability and pulmonary edema in ALI. Meanwhile, the increased apoptosis of alveolar epithelial cells could lead to a decrease in alveolar surfactants, resulting in alveolar collapse and atelectasis [8,9]. Although the pathogenesis of ALI is not fully understood, multiple studies elucidated that the apoptosis of alveolar epithelial cells plays a key role [10,11,12]. Therefore, it is particularly important to explore a way to reduce apoptosis of alveolar epithelial cells.

The heat shock protein family (HSPs) is a class of highly conserved proteins widely found in various organisms. Among all HSPs, HSP70 is known as major heat shock proteins, including inducible and structural forms. Inducible HSP70, not structural HSP70 (HSC70), is usually lower in normal cells, but its expression increases rapidly under heat stimulation or other stress [13]. HSP70 is edited by two 99% homology genes of *hsp70.1* and *hsp70.3* in mice. The *hsp70.1* gene is the most consistently induced *hsp70* gene response to stress [14]. The research has reported that HSP70 plays a crucial role in protein assembly, folding, and transport [15,16,17,18]. Indeed, studies have shown that HSP70 protects cells from thermal oxidation, mechanical damage, and inflammation by interfering with apoptosis at multiple levels. In homozygous HSP70 knockout mice (*hsp70.1*^−/−^), apoptosis of bronchiolar epithelial cells was significantly increased in the chronic hypoxia mice model [19]. Kondrikov’s study showed that overexpression of HSP70 prevented hyperoxia-induced apoptosis in pulmonary artery endothelial cells [20]. Given the plethora of effects attributed to inhibition of apoptosis, it is not clear whether this benefit can be ascribed to the protective effect and what is the molecular mechanism for systemic application of recombinant HSP70 in cecal ligation and puncture (CLP)-induced septic lung injury.

Therefore, the broad aims of this study were to evaluate the protective effect of HSP70 on survival, lung injury, and apoptosis following sepsis induced by CLP in mice and LPS stimulation in alveolar epithelial cells. In addition, we investigated the mechanisms underlying this, utilizing overexpression of HSP70 cells by RNA sequencing, mass spectrometry, and Co-IP. Knockdown of KANK2 in cells and *hsp70.1* gene knockout mice as well as their wild-type counterparts with CLP were used to verify whether KANK2 is one of the binding proteins with HSP70 for alleviating pulmonary dysfunctions in septic lung injury.

## 2. Materials and Methods

### 2.1. Animals and Sepsis Model

A total of 156 C57BL/6 male mice (6–8 weeks old, weighing 20–25 g, 6–8 per group) were purchased from Shanghai Laboratory Animal Center, Chinese Academy of Sciences. The experiment was conducted one week after the mice adapted to the new environment. HSP70 (*hsp70.1*) knockout C57BL/6 mice were generated by Cyagen Biosciences Inc. (Guangzhou, China) through CRISPR/Cas9-mediated genome editing (serial number: KOCMP-15511-hsp70.1). The animal experiments were under the guideline of the Animal Research Committee of the Shanghai Jiao Tong University School of Medicine.

Sepsis was induced by CLP. The mice were anesthetized by intraperitoneal injection of 1% pentobarbital (10 mL/kg), and the abdomen was depilated and disinfected with povidone iodine. The cecum was exposed and a ligature was performed on the first 1/3 of the cecum. Two cecal punctures were made with a 22-gauge needle, and a small amount of intestinal contents was pressed out. The bowel loops were returned to the abdominal cavity, and the wound was closed in layers using 5–0 surgical sutures (BD, Franklin Lakes, NJ, USA). Sham-operated mice were operated on using the same operation methods, except that the cecum was not ligated or punctured. Before and after the surgery, animals had free access to food and water. HSP70-treated mice were intravenously injected with recombinant human HSP70 (AP-100–100, Bostonchem, Boston, MA, USA) at a concentration of 10, 50, and 250 μg/kg. Twenty-four hours after surgery, the gross profile was observed and sepsis score was assessed according to the Modified Murine Sepsis Score (MSS) [21]. Then, the mice were killed and lungs were removed for further evaluation. The wet/dry ratio was calculated by measuring wet and dry weight of the lung. The remaining lung tissue was fixed for paraffin section or stored in −80 °C refrigerator for further RNA or protein extraction. For HSP70 knockout experiments, mice were divided into three groups (eight per group) with CLP: *hsp70.1*^+/+^, *hsp70.1*^+/−^, *hsp70.1*^−/−^. The gross profile was observed and the lungs were collected for further evaluation.

### 2.2. Enzyme-Linked Immunosorbent Assay

The levels of serum TNF-α and IL-6 were detected by ELISA. Blood samples from mice were collected and centrifuged at 3000 rpm 4 °C for 10 min. The supernatant serum was analyzed, utilizing ELISA kits for mice TNF-α and IL-6 (Cat#MTA00B, Cat#M6000B, R&D, Minneapolis, MN, USA), following the instructions. Results were determined using Infinite M200pro spectrophotometer (Tecan, Männedorf, Switzerland).

### 2.3. Cell Culture and Cell Treatment

Human alveolar epithelial cells (A549) and human embryonic kidney (HEK) 293T cells were purchased from Cell Resource Centre of Shanghai Institute for Biological Science, Chinese Academy Science. Cells were cultured in RPMI-1640 medium (HyClone, Logan, UT, USA), supplemented with 10% fetal bovine serum, 1% penicillin G, and 1% streptomycin (Gibco, San Diego, CA, USA). The cells were maintained at 37 °C in a humidified atmosphere containing 5% CO_2_. To induce apoptosis, the cells were treated with LPS (Sigma Aldrich, St. Louis, MO, USA) at a concentration of 4 µg/mL for 24 h (unless specified). The HSP70 was diluted into a concentration of 0.05, 0.2, and 1 μg/mL for use, and was applied to the A549 cells 15 min before LPS stimulation. To detect whether HSP70 was uptaken, A549 cells were treated with 0.2 and 1 μg/mL HSP70 and collected at 5, 15, 30, 60, and 180 min for WB.

### 2.4. Lentivirus Transduction and Cell Transfection

The plasmid pCDNA3.1-Flag-HSP70 was obtained from Public Protein/Plasmid Library (Nanjing, China) and the plasmid pCMV-HA Vector was from Clonetech Laboratories, Inc. Those plasmids were used for protein expression. The pCMV-HA-KANK2 Vector was constructed by inserting human *kank2* into the pCMV-HA Vector. HEK 293T cells were transfected with plasmids using FuGENE 6 Transfection reagent (Promega, Madison, WI, USA) for 24 h. To establish stable HSP70-overexpressing A549 cells (HSP^OE^ A549 cells), lentiviral of pCD513B containing *hsp70* was utilized and 10 μg/mL puromycin (Gibco, San Diego, CA, USA) was applied post infection. The detailed information was described previously [22]. The HSP70 gene knockdown A549 cells mentioned in this study were constructed using the short hairpin RNA (shRNA) targeting HSP70 according to the manufacturer’s instructions (GenePharma, Shanghai, China). The targeting sequence was 5′-GGUCCUAAGAAUCGUUCAATT-3′. The si-NC and si-KANK2 were synthesized by RiboBio Co. (Guangzhou, China). The sequences for KANK 2 siRNA were: 5′- TCGAGAATCTCAGCACATA-3′. The siRNAs were applied to A549 cells according to the instructions at 50 nM.

### 2.5. Cell Viability by Cell-Counting Kit (CCK)-8

Cell viability was evaluated by CCK-8. A549 cells were seeded in 96-well plates at 5000 cells/well. Cells were cultured with LPS, with/without HSP70, si-NC or si-KANK2 for 24 h. Next, 10 µL of the CCK-8 solution (Dojindo, Tokyo, Japan) was added to each well and incubated for 1 h at 37 °C. The OD value at 450 nm was measured by Infinite M200pro spectrophotometer (Tecan, Männedorf, Switzerland). Each group had six replicates and the experiment was repeated three times. The cell viability was determined according to the following formula:

Cell viability % = (OD_experimental_ − OD_blank_)/(OD_ctrl_ − OD_blank_) × 100.

### 2.6. Clone Formation Assay

For the assay, 800 cells were seeded in six-well plates per well. After adherence, cells were treated with LPS, with/without HSP70, si-NC, or si-KANK2 and cultured for 7 days. Then, cells were fixed with 4% paraformaldehyde and stained with crystal violet (Beyotime, Shanghai, China) for 10 min. Clones that contained in excess of 50 cells were counted as one colony and the images were taken under a microscope in the low-power field of view (Carl Zeiss, Jena, Germany). The clone formation efficiency was calculated and analyzed.

### 2.7. Flow Cytometry

The A549 cells were planted in six-well plates and treated as indicated. After 48 h, the cells were collected, centrifuged at 800 rpm for 5 min, and washed with PBS twice. A volume of 100 μL 1× Annexin V binding buffer was added to resuspend the cells to adjust the cell density at 1 × 10^6^ cells/mL. Then, the cells were stained with Annexin-V conjugated FITC and PI kit (Dojindo, Tokyo, Japan). Flow cytometry detection was performed using Beckman CytoFlex S (Beckman Coulter, Fullerton, CA, USA), and data was analyzed using CytExpert software. The experiment was repeated three independent times.

### 2.8. Western Blotting

Proteins from cells or lungs were extracted using RIPA Lysis Buffer with Complete Protease Inhibitor Cocktail (Cell Signaling, Danvers, MA, USA). To detect the release of AIF from mitochondria, cells or tissue mitochondria isolation kit (Beyotime, Shanghai, China) were used to separate the protein, and cytoplasm (without mitochondria fractions) was collected for WB. WB was performed as described previously [23]. The protein levels were measured with specific primary antibodies including HSP70 (ab79852, Abcam, Cambridge, UK), KANK2 (PA5-116620, Invitrogen, Waltham, MA, USA), Bax (ab32503, Abcam, Cambridge, UK), Bcl-2 (ab194583, Abcam, Cambridge, UK), GAPDH (9485, Abcam, Cambridge, UK), β-actin (ab8226, Abcam, Cambridge, UK), and AIF (4642, Cell Signaling, Danvers, MA, USA) at a dilution of 1:1000. The appropriate secondary antibodies were chosen to incubate with membrane for 2 h at room temperature. Finally, protein bands were applied with enhanced chemiluminescence detection kit (Thermo Scientific, Portsmouth, NH, USA) and imaged by fusion-capture software (Fusion FX7, Marne-la-Vallée, France).

### 2.9. Immunoprecipitation

Cells were lysed with RIPA buffer. After quantification, the proteins were incubated with protein A/G agarose beads (Santa Cruz, Dallas, TX, USA) and antibodies against IgG (2729, Cell Signaling, Danvers, MA, USA), Flag (F3165, Sigma Aldrich, St. Louis, MO, USA), or HSP70 (ab5442, Abcam, Cambridge, UK). For IP Flag-HSP70, protein was incubated with mouse anti-Flag M2 protein A/G agarose beads (Sigma Aldrich, St. Louis, MO, USA) overnight at 4 °C. After washing, immunoprecipitates were collected and analyzed by WB or for protein profile and MS.

### 2.10. Quantitative PCR

Total RNA of lungs and cells was extracted and reversed using Trizol and FastKing RT Kit (Tiangen Biotech, Beijing, China). The qPCR was performed using the SYBR Green RT-PCR Kit (Qiagen, Hilden, Germany) and the Light Cycler 480 Real-Time PCR Instrument (Roche, Basel, Switzerland). The primer sequences were synthesized by Shanghai Sangon Biotech as follows: GAPDH forward 5′-ATGGGGAAGGTGAAGGTCG-3′ and reverse, 5′-TAAAA GCAGCCCTGGTGACC-3′; IL-6 forward 5′-CACATGTTCTCTGGGAAATCGTGGA-3′ and reverse 5′- TCTCTCTGAAGGACTCTGGCTTTGT-3′; TNF-α forward 5′- CCCTCAC ACTCAGATCATCTTCT-3′ and reverse 5′-GCTACGACGTGGGCTACAG-3′.

### 2.11. TdT-Mediated dUTP Nick End Labelling (TUNEL) Staining

Apoptosis in lung tissue was detected and quantified by the TUNEL assay using the In Situ Cell Death Detection Kit (Roche, Mannheim, Germany, TMR red) according to the protocol. Apoptotic cells exhibited red staining in the cell nuclei. The number of TUNEL-positive cells in random field of view was calculated and analyzed.

### 2.12. RNA Sequencing

RNA from Flag-HSP70 HEK 293T cells and control was extracted with Trizol. The transcriptome sequencing experiment process includes RNA extraction, RNA sample quality detection (Agilent Bioanalyzer 2100, Santa Clara, CA, USA), library construction (NEBNext Ultra RNA Library Prep Kit for Illumina, Ipswich, MA, USA), library purification, library detection, library quantification (Agilent Bioanalyzer 2100, Santa Clara, CA, USA and Qubit, Waltham, MA, USA), sequencing cluster generation (TruSeq PE Cluster Kit, San Diego, CA, USA), and then computer sequencing (TruSeq SBS Kit, San Diego, CA, USA). After obtaining the original sequencing data (Pass Filter Data), bioinformatics analysis is performed by GeneWiz (Suzhou, China).

### 2.13. Silver Stain and Mass Spectrometry

Proteins from HEK 293T cells transfected with Flag-HSP70 were extracted and immunoprecipitated by anti-IgG/anti-Flag together with protein A/G agarose beads. Immunoprecipitates were separated by SDS-PAGE. The Fast Silver Stain Kit (Beyotime, Shanghai, China) was used for the staining following the instruction and protocol described [24]. Flag-HSP70 pulldown band and IgG pulldown band were cut for mass spectrometry. The target band was enzymatically hydrolyzed at 37 °C for 20 h and then peptides were separated with Easy nLC (Thermo Scientific, Portsmouth, NH, USA), and followed by analysis using Q-Exactive mass spectrometer (Thermo Scientific, Portsmouth, NH, USA). Twenty fragment maps were collected after each full scan for the mass-to-charge ratio of the polypeptide and the fragment. Data were analyzed using Mascot 2.2 software. The bioinformatics analysis of mass spectrometry was completed by Shanghai Applied Protein Technology Co., Ltd. (Shanghai, China).

### 2.14. Immunofluorescence, Hematoxylin and Eosin (H&E) Staining, and Immunohistochemistry

The expression of HSP70 and KANK2 in A549 cells was detected by IF using mouse HSP70 (ab5442, Abcam, Cambridge, UK, 1:200), rabbit KANK2 (SAB4501100, Sigma Aldrich, St. Louis, MO, USA, 1:100), anti-rabbit IgG Alexa Fluor 647 (A-31573, Invitrogen, Waltham, MA, USA, 1:500), and anti-mouse IgG Alexa Fluor 488 (ab150113, Abcam, Cambridge, UK, 1:500) antibodies following the previous protocol [22]. Images were analyzed through a Leica-SP8 confocal microscope (Leica, Wetzlar, Germany). Mice lung specimens were paraffin embedded and cut (5 μm) for HE and IHC staining. The histological score of lung tissue was evaluated by a pathologist blindly according to the severity of inflammation within the tissues [25]. The expression of KANK2 was measured by IHC with primary anti-KANK2 antibody (PA5-116620, Invitrogen, Waltham, MA, USA, 1:100). The photographs were taken under Nikon Ti-S microscope (Tokyo, Japan) and area of positive cells was analyzed by Fiji image processing software.

### 2.15. Mitochondrial Membrane Potential Detection

Mitochondrial membrane potential (MMP) was measured by Mito-Tracker Red CMXRos (Invitrogen, Waltham, MA, USA). For the assay, A549 cells were seeded in 12-well plates. After adherence, cells were treated with LPS, si-NC, or si-KANK2 and cultured for 48 h. Then, the medium was removed and mito-Tracker Red CMXRos working solution was added. After incubating at 37 °C for 30 min, the working solution was removed and replaced with fresh medium. The fluorescence intensity was measured by imaging (Ex 579 nm, Em 599 nm) using the IVIS Spectrum CT imaging system (PerkinElmer, Waltham, MA, USA).

### 2.16. Statistical Analysis

Data are represented as mean ± SD. Statistical analysis of the data was performed by one-way analysis of variance or Student’s *t*-test using SPSS 25 and GraphPad Prism 7 software. All experiments were repeated at least three times and *p* < 0.05 was considered to be statistically significant.

## 3. Results

### 3.1. HSP70 Alleviates Pulmonary Functions in Septic ALI through Reducing Apoptosis In Vivo and In Vitro

**Figure 1:**
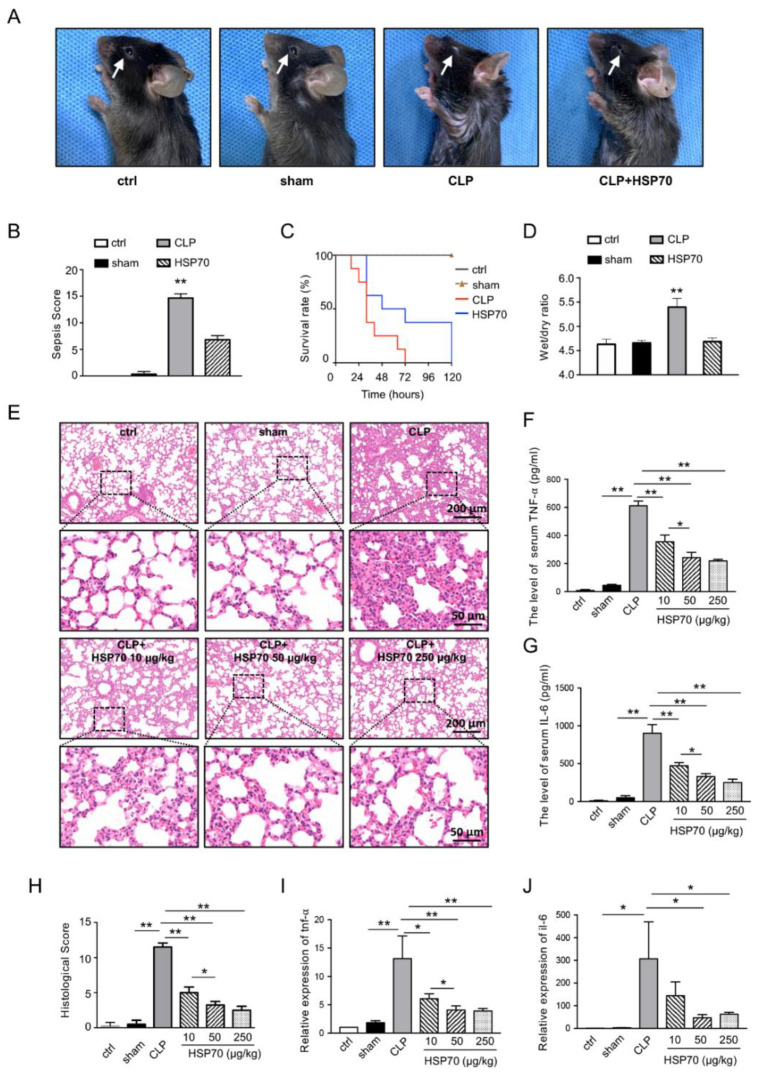
HSP70 improves septic lung functions and reduces inflammation. C57BL/6 mice treated with CLP and HSP70 (50 µg/kg or as indicated) were assessed for the gross profile (white arrow indicated the secretions of mouse eyes) (**A**), sepsis score (**B**), survival rate (**C**), and wet/dry ratio (**D**). The morphological changes of lung tissue were observed by H&E staining and scores of lung injury were evaluated (**E**,**H**). The levels of serum TNF-α (**F**) and IL-6 (**G**) were measured by ELISA. The mRNA levels of *tnf-α* (**I**) and *il-6* (**J**) in lung tissues were measured using qPCR. Data are presented as mean ± SD of data from three independent experiments. * *p* < 0.05, ** *p* < 0.01.

To evaluate the effects of HSP70 on septic ALI, sepsis mice model by CLP was established and treated with 50 μg/kg of HSP70. The gross profile of animals was observed using sepsis scoring system. The mice in CLP group were in poor condition, with ruffled fur, lower level of consciousness, reduced activity, weak response to stimulus, and mostly closed eyes with secretions at the corners of their eyes while the sham mice were totally recovered from surgery at 24 h. With HSP70 treatment, the general conditions of CLP mice were much healthier and improved (Figure 1A). The sepsis score of mice was evaluated (Figure 1B) and there were significant differences among those groups (*p* < 0.01). Additionally, the survival rate of mice showed that CLP mice started to die by 16 h and 100% of CLP mice died by the end of 72 h, compared with 0% of sham mice dying by 120 h. The mice in the CLP plus HSP70 group began to die by 32 h, and the survival rate was near 40% at 72 h. All the mice died after 120 h. The survival rate in CLP mice was significantly lower than that in the CLP plus HSP70 group (Figure 1C, *p* < 0.05). Meanwhile, the wet/dry ratio of lung in the CLP group was 5.39 ± 0.18, which was higher than that in the control and sham group. After treatment with HSP70, the increased wet/dry ratio by CLP was decreased to 4.68 ± 0.07 (*p* < 0.01, Figure 1D). In addition, the H&E section of lung from the sham group had complete alveoli, clear structure, and was basically close to normal. The lung histology in the CLP showed that a large number of inflammatory cells infiltrated the interstitium, alveolar capillary membrane was thickened, and the alveolar structure was destroyed and fused. Some red blood cells and neutrophils were seen in the alveolar cavity. After HSP70 treatment, the mice lung damage was dramatically alleviated (Figure 1E,H). Besides, the level of serum TNF-α and IL-6 (Figure 1F,G) and their gene expressions in the lung tissue (Figure 1I,J) of CLP mice were significantly higher than those in the sham group and the HSP70-treated group.

**Figure 2:**
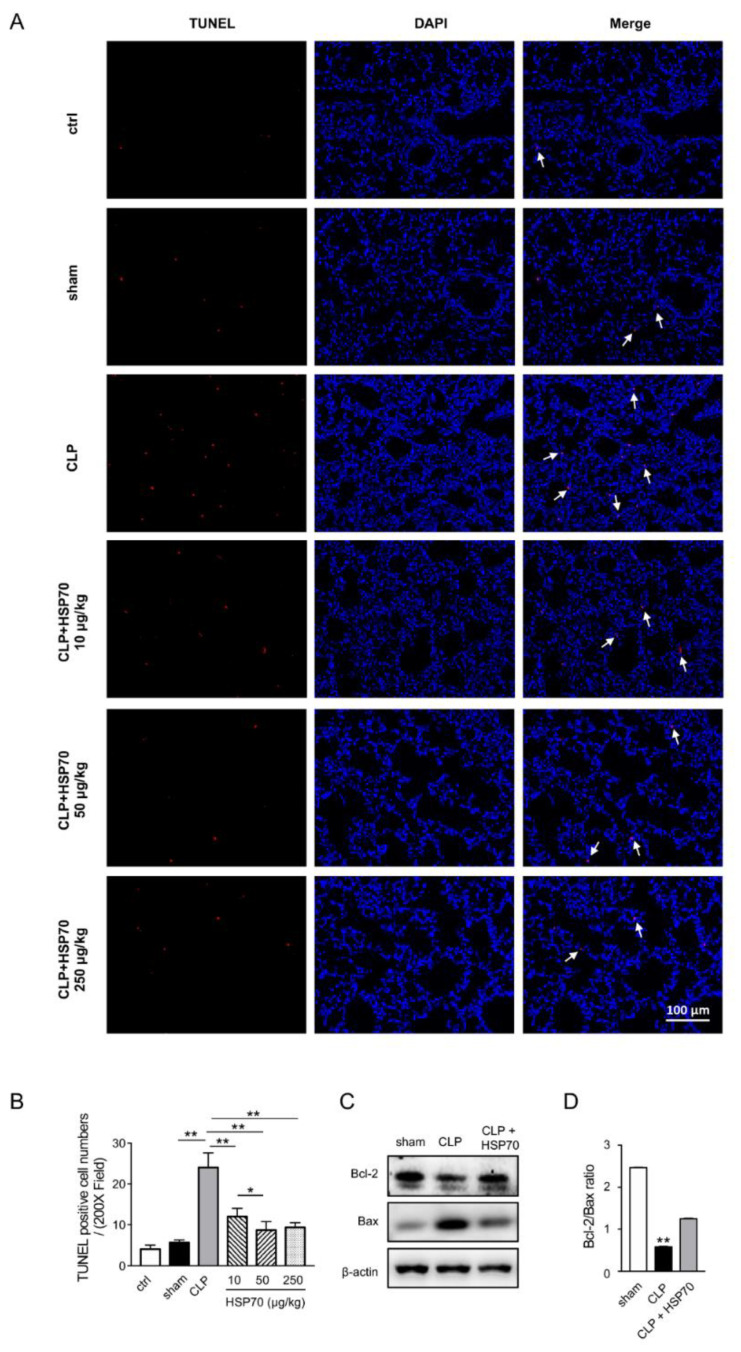
HSP70 reduces apoptosis in septic lung injury. C57BL/6 mice were treated with CLP and HSP70 (50 µg/kg or as indicated) and the lung tissues were collected. TUNEL staining showed the dead cells of lung tissue as indicated with white arrow. TUNEL-positive, red; DAPI, blue (**A**,**B**). The levels of Bax and Bcl-2 were measured using WB (**C**,**D**). Data are presented as mean ± SD of data from three independent experiments. Bar = 100 µm. * *p* *<* 0.05, ** *p* < 0.01.

We further observed the apoptosis in lung tissues using TUNEL staining. Compared with that in controls and shams, far more TUNEL-positive cells were seen in the lung of CLP mice. The increased positive cells in CLP mice were reduced by treatment with HSP70 (Figure 2A,B, *p* < 0.05). Moreover, the levels of apoptotic-related proteins Bax and Bcl-2 in the lung tissue were detected by WB. The level of Bax was increased and Bcl-2 was reduced remarkably in CLP group compared to that in the sham and HSP70-treated mice group (Figure 2C,D). Those results suggest that HSP70 improves the function of septic lung injury that is caused by inflammation and apoptosis in vivo.

**Figure 3:**
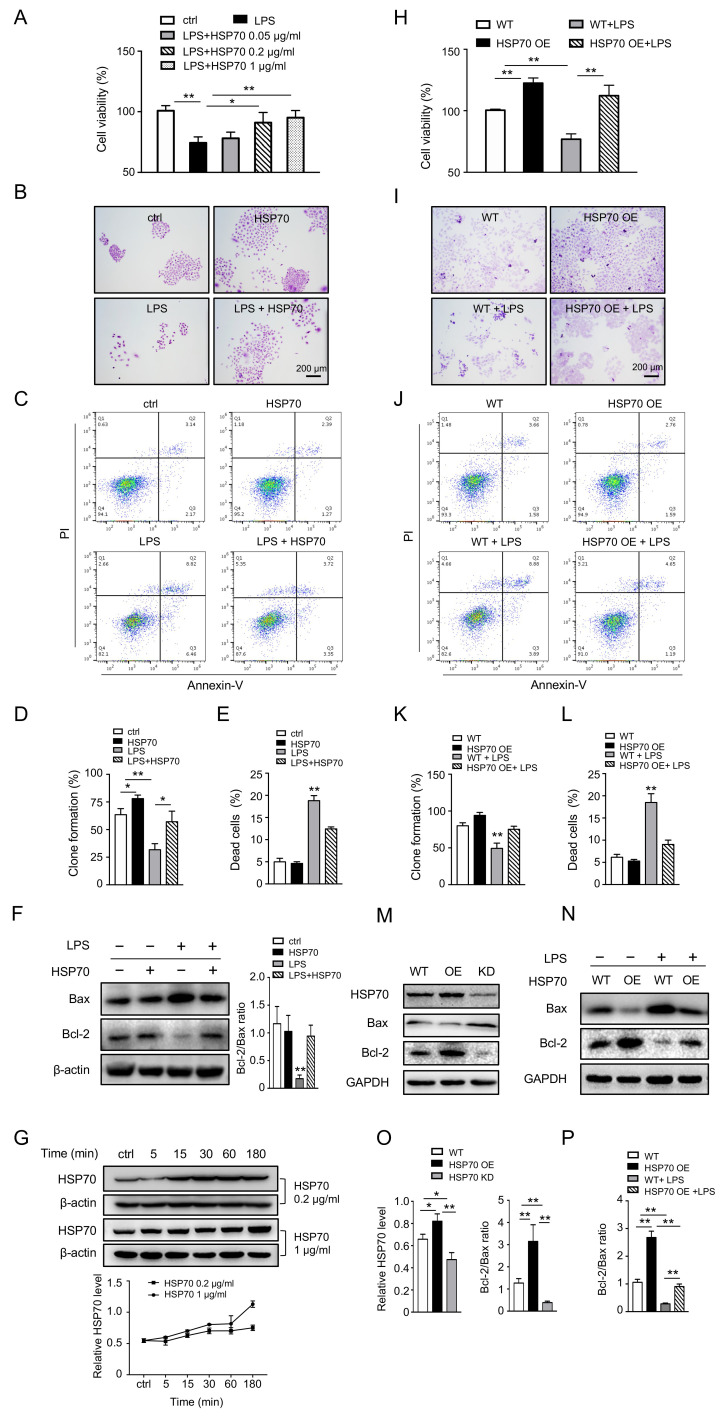
Effects of HSP70 on apoptosis in alveolar epithelial cells. Human alveolar epithelial A549 cells were cultured and treated with LPS (4 µg/mL) and HSP70 (0.2 µg/mL was used unless specified). Cell viability (**A**) was measured by CCK-8 and cell colony formation (**B**,**D**) was evaluated by crystal violet staining. Dead cells were enumerated by flow cytometry labelling with Annexin V and PI (**C**,**E**). The levels of Bax and Bcl-2 were measured using WB (**F**). The levels of HSP70 in A549 cells treated with 0.2 and 1 µg/mL HSP70 at different time points were measured using WB (**G**). HSP70^OE^ A549 cells were established and treated with LPS. Cell viability (**H**), cell colony formation (**I**,**K**), and dead cells (**J**,**L**) were measured. The levels of HSP70, Bax, and Bcl-2 were measured using WB (**M**–**P**), including the level in HSP70 knockdown A549 cells. Data are presented as mean ± SD of data from three independent experiments. Bar = 200 µm. * *p* < 0.05, ** *p* < 0.01.

To verify these in vivo results, alveolar epithelial A549 cells were treated with LPS to mimic pulmonary cell injury in septic animal model and were then treated with/without HSP70. Cell viability and clone formation were significantly reduced by LPS (*p* < 0.01), compared to those in the controls. Those reductions were restored by HSP70 intervention (Figure 3A,B,D, *p* < 0.05). Furthermore, the cell apoptosis was determined by flow cytometry with Annexin V and PI labelling. The proportion of dead cells in A549 treated with LPS (18.80 ± 0.67%) was much higher than that in the control (5.00 ± 0.79%) and the HSP70 only group (4.63 ± 0.37%, *p* < 0.01). However, LPS-induced apoptotic cells were reversed by HSP70 treatment (12.43 ± 0.45%, Figure 3C,E, *p* < 0.01). Meanwhile, we found a very similar pattern of apoptotic-related protein levels (Bax, Bcl-2) that we have seen in vivo (Figure 3F). Besides, the levels of intracellular HSP70 after applying the recombinant HSP70 to the medium of A549 cells were detected. The HSP70 increased from 5 min to 15 min and stayed for more than 180 min (Figure 3G, *p* < 0.01). In addition, HSP70^OE^ A549 cells were used to evaluate the effect of HSP70. Consistently, the results in HSP70^OE^ A549 and A549 cells treated with HSP70 came to the same conclusion (Figure 3H–P). Our data demonstrate that HSP70 plays remarkable roles in protecting the lung from septic injury both in vivo and in vitro.

### 3.2. HSP70 Interacts with KANK2 to Prevent Epithelial Cells from Apoptosis In Vitro

**Figure 4:**
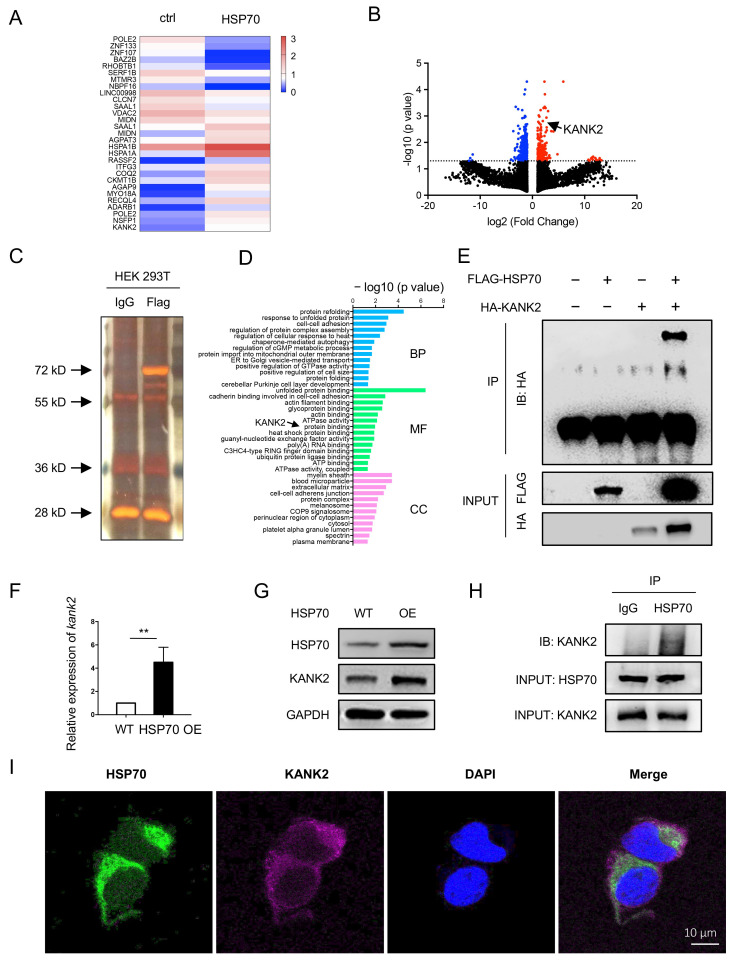
RNA sequencing and proteomics in HEK 293T cells and validation in A549 cells. HEK 293T cells were transfected with *hsp70*-plasmid. Transcriptomes analysis was conducted in HEK 293T cells by RNA sequencing. Heat map of the cluster analysis (**A**) and volcano plot (**B**) was presented. The protein binding to HSP70 was extracted by IP (anti-Flag-HSP70). Then, the protein was separated by electrophoresis and stained with silver (**C**). Proteomics analysis was conducted by MS. Enrichment analysis for the binding protein was shown (**D**). IP was performed with an anti-Flag antibody. The expressions of HA-KANK2 and Flag-HSP70 were measured in HEK 293Ts. KANK2 bands were indicated with black arrow (**E**). A549 cells were cultured. The mRNA (**F**) and protein (**G**) levels of KANK2 in HSP^OE^ A549 cells were measured using qPCR and WB. IP was performed with an anti-HSP70 antibody, and KANK2 was assessed in A549 cells (**H**). Representative immunofluorescence images of HSP70 and KANK2 in the A549 cells were acquired (**I**). Bar = 10 µm. BP, biological process; CC, cellular component; MF, molecular function; MS, mass spectrometry. ** *p* < 0.01.

To investigate the mechanisms underlying the HSP70 effects on septic ALI, further experiments were performed in various cells. The HEK 293T cells overexpressed with/without HSP70 were analyzed by RNA sequencing. A total of 507 differential transcriptomes were identified according to the criteria of *p* value < 0.05 and an absolute log2 (fold change) > 1. The heat map revealed the substantial differences in the two indicated groups (Figure 4A). The volcano plot showed 18,981 of all transcriptomes (absolute log2 (fold change) > 1, indicated by dots), red dots indicated upregulated and blue dots were downregulated (Figure 4B). Among which the *kank2* was remarkably upregulated (*p =* 0.00155) by HSP70. Furthermore, the protein binding to HSP70 was extracted from overexpressed Flag-HSP70 HEK 293T cells with anti-Flag antibody by IP. Then, the proteins that included anti-IgG and anti-Flag were separated for protein profiling (Figure 4C). By mass spectrometry, there were more than 120 proteins or fragments that bind to HSP70, involving protein folding, autophagy, protein binding, cell membrane components, and so on (Figure 4D). Intriguingly, KANK2 was one of 120 proteins. Our data revealed that KANK2 interacted with HSP70. To confirm this, Flag-HSP70 and HA-KANK2 were co-expressed in HEK 293T cells and then isolated with anti-Flag beads by IP to assess HSP70-KANK2 binding. An HA-KANK2 band in the Flag-HSP70 was seen in the blotting (Figure 4E). Subsequently, we confirmed the results of HEK 293T cells using A549 cells by HSP70 overexpression. The mRNA and protein level of KANK2 in the HSP70^OE^ A549 cells was much higher than that in the controls (Figure 4F,G). The HSP70-KANK2 binding was detected by co-IP with an anti-KANK2 antibody in A549 cells (Figure 4H). Moreover, the merged image of HSP70 (green) and KANK2 (purple) in the cytoplasm was observed by IF (Figure 4I), supporting the interaction between HSP70 and KANK2. Taken together, these results demonstrate that KANK2 can bind to HSP70. Thus, we speculate that KANK2 is critical in HSP70 protection of septic ALI.

**Figure 5:**
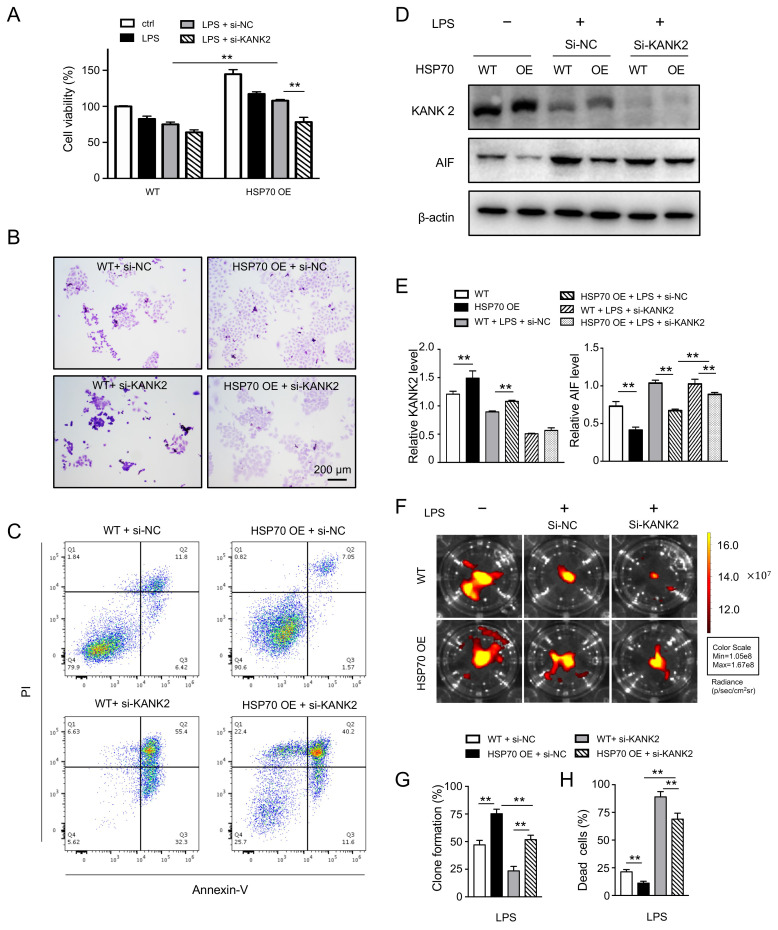
Interaction of HSP70 and KANK2 in A549 cells. WT and HSP70^OE^ A549 cells were treated with LPS (4 µg/mL) and si-KANK2 (50 nM, si-NC was set as corresponding control). Cell viability (**A**) was measured by CCK-8 and cell colony formation (**B**,**G**) was evaluated by crystal violet staining. Dead cells were enumerated by flow cytometry labelling with Annexin V and PI (**C**,**H**). The levels of KANK2 and released AIF were measured using WB (**D**,**E**). Mitochondrial membrane permeability was assessed by mito-tracker and fluorescence intensity was measured by imaging system (**F**). Data are presented as mean ± SD of data from three independent experiments. Bar = 200 µm. ** *p* < 0.01.

To determine whether HSP70 inhibits the apoptosis of A549 cells through KANK2, we assessed effects of HSP70 in cells after interrupting KANK2. The restorations of cell viability (Figure 5A) and clone formation (Figure 5B,G) by LPS plus HSP70 were significantly reduced after si-KANK2 intervention in A549 cells. The proportion of dead cells in HSP OE si-KANK2 group (68.93 ± 9.55%) was much higher than that (11.17 ± 0.98%) in the si-NC group (Figure 5C,H). The decreased apoptotic cells were increased again, suggesting KANK2 plays a critical role in protection of HSP70. It has been reported that KANK2 inhibits apoptosis by preventing the release of AIF from mitochondria into the nuclei [26]. To make it clear, cell cytoplasm fractions without mitochondria were collected and the level of AIF treated with/without si-KANK2 was checked. Compared with that in the si-NC group, the amount of AIF released from mitochondria of HSP^OE^ A549 cells was much higher in the si-KANK2 group (Figure 5D,E). Since the mitochondrial membrane permeability decrease could cause the release of AIF, the lower fluorescence of mito-tracker indirectly reflected that AIF increased after inhibition of KANK2 (Figure 5F). The data demonstrate that the protection of KANK2 from apoptosis is accomplished through mediating AIF.

### 3.3. Interaction of KANK2 and HSP70 Inhibits Apoptosis and Ameliorates Septic Lung Function In Vivo

**Figure 6:**
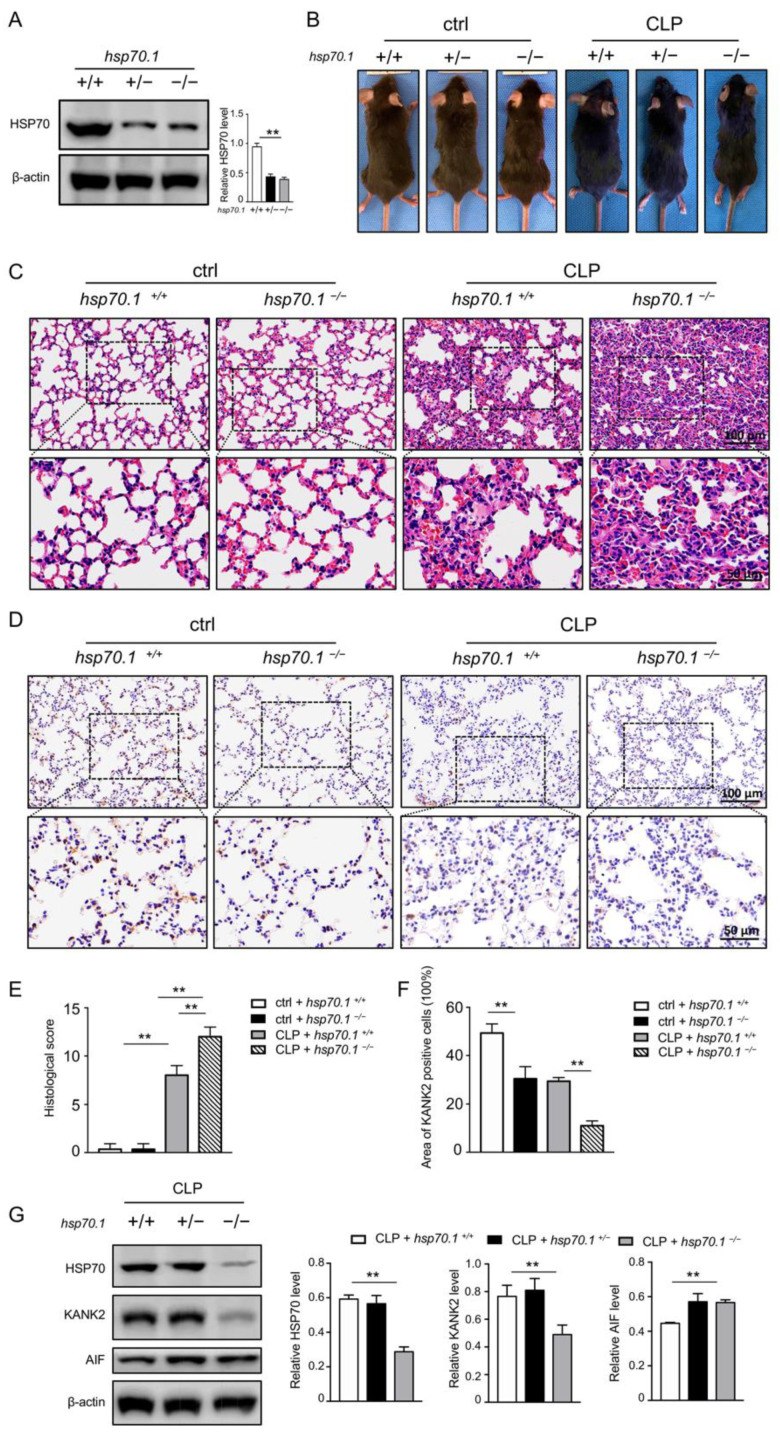
Interaction of HSP70 and KANK2 in *hsp70.1*^−/−^ mice. *hsp70.1*^+/+^, *hsp70.1^+/−^*, and *hsp70.1*^−/−^ mice were bred and the lung tissues were collected. The levels of HSP70 were measured in mice lung by WB (**A**). Gross profile of mice before and after CLP was assessed (**B**). The pathological appearances of lung tissue were evaluated by H&E staining (**C**,**E**). The KANK2-positive cells were evaluated by IHC. Brown color indicates positive staining of KANK2 (**D**,**F**). The levels of HSP70, KANK2, and released AIF were measured using WB (**G**). Data are presented as mean ± SD of data from three independent experiments. ** *p* < 0.01.

To verify the above results, *hsp70.1*^−/−^ mice were utilized for creating septic model by CLP. The levels of HSP70 were measured by WB. The total HSP70 decreased in *hsp70.1*^−/−^ mice (Figure 6A). Gross observations of *hsp70.1*^−/−^ mice were presented (Figure 6B). Compared to *hsp70.1*^+/+^ mice, *hsp70.1*^−/−^ mice were prone to CLP injury, including more ruffled fur and reduced activity, lower level of consciousness, worse response to stimulus, mostly closed eyes with secretions at the corners of their eyes, and lower survival rate (unpublished data) [27]. The H&E staining in both *hsp70.1*^+/+^ mice and *hsp70.1*^−/−^ mice showed a normal pulmonary view (Figure 6C,E). At 24 h post CLP, pulmonary histology in CLP animals presented an excessive interstitial edema, thickened alveolar walls, and numerous infiltrations of inflammatory cells. The histologic image in *hsp70.1*^−/−^ mice suffering with CLP showed even worse pathologic change. Additionally, a lower level of KANK2 was seen in *hsp70.1*^−/−^ mice, compared to that in the *hsp70.1*^+/+^ mice by HIS (Figure 6D,F). The level of released AIF in *hsp70.1*^−/−^ mice suffering CLP was higher than that in *hsp70.1*^+/+^ mice with CLP (Figure 6G). These results indicate that HSP70 could intermingle with KANK2 to prevent AIF translocation, inhibit apoptosis, and protect septic lung injury.

## 4. Discussion

The data presented here demonstrate that the treatment of HSP70 significantly alleviated pulmonary functions and inhibited AIF-induced apoptosis after a septic lung injury. Furthermore, that interaction of KANK2 and HSP70 was critical for HSP70 to protect against septic lung injury. The findings of this study elucidate potential therapeutics by which heat stress proteins can protect lung function from septic ALI.

Members of the HSP family, especially the inducible HSP70 [28], play pivotal roles in cell death and are associated with sepsis [29]. However, the function of recombinant HSP70 in septic ALI was not fully understood. In this study, we showed that the survival rate, histologic pulmonary damage, cell viability, and apoptosis caused by sepsis or LPS stimulation were attenuated by treatment of recombinant HSP70 and in HSP70-overexpressing cells. Taken together, we demonstrated that HSP70 was responsible for suppressing cellular apoptosis and was able to inhibit lung tissue damage and increase survival rate in ALI caused by sepsis. Additionally, when the gene for HSP70 was deleted, these effects were reversed. Our results suggest that the protection from injury that is conferred by exogenous HSP70 acts at least partly through the apoptotic pathway. Interestingly, HSP90, as one other chaperone protein from HSP families, has been reported to be upregulated in various diseases, including cancer and sepsis [30,31]. HSP90 is responsible for tertiary folding of client proteins such as IKK. The folding function of HSP90 acts downstream of HSP70 in protein folding without further co-chaperones or with co-chaperones such as p23 and Cdc37 [32]. The binding of HSP90 protein and its co-chaperone Cdc37 to the IKK complex is required for the production of proinflammatory cytokines [33]. However, HSP70 protein binds to the coiled-coil domain of NEMO and the binding impedes the formation of active IKK complexes, inhibiting NF-κB activation [34]. 17-dimethylaminoethylamino-17-demethoxygeldanamycin (17-DMAG), an inhibitor which attenuates the ATPase activity of HSP90, was reported to possess anti-inflammatory effects, associated with activation of heat shock factor (HSF)-1, leading to induction of HSP70 production, ameliorating multiple organ dysfunction syndrome in septic rats, and preventing lipopolysaccharide-induced liver injury in mice [35,36]. The interaction of HSP70 and HSP90 in septic lung injury in our model needs to be further explored. Multiple studies have shown that different chemical stimulations, such as geranylgeranylacetone, selective κ-opioid receptor agonist U-50488H, anandamide, candesartan, and adrenomedullin, can induce the secretion of HSP70 [37,38,39,40,41,42]. However, the levels of HSP70 with different stimulations were varied greatly, which resulted in a highly controversial outcome of treatment. As HSP70 could be released and uptaken by various cell types, extracellular HSP70 could bind to a wide repertoire of cell-surface receptors that may result in internalization and intracellular responses [43,44,45]. Thus, it could be considered to be a promising way to apply exogenous HSP70 for protection in the current study.

However, the mechanism for the protective effect of HSP70 in septic ALI has not been fully studied. Although our study and previous studies suggest that HSP70 inhibits the secretion of inflammatory mediators, such as TNF-α, which alleviate alveolar epithelial injury to a certain extent, the direct protective effect of HSP70 on alveolar epithelial cells needs to be further explored. HSP70 is a classical molecular chaperone and contains a highly conserved N-terminus (ATP binding domain) and a C-terminus (substrate binding domain) with a molecular weight of about 44 kD and 25 kD, respectively. In the state of stress, the binding of HSP70 to other proteins can prevent the denaturation or depolymerization of the proteins. Considering those, we speculate that HSP70 may act through direct or indirect binding to a certain protein. We utilized overexpressed cells of HSP70 and *hsp70.1*^−/−^ CLP mice for research. RNA-seq and mass analyses revealed that KANK2 is one of the proteins interacting with HSP70 for exerting biological activity. The overexpression of HSP70 interfered with apoptosis, specifically, through reducing mitochondrial release of AIF. KANK2 is called steroid receptor coactivator (SRC)-interacting protein (SIP), and is characterized by an N-terminal KN motif, coiled-coil domains, and C-terminal ankyrin repeat domains [46]. KANK2 interacts with SRC co-activators in the cytoplasm and regulates the transactivation activity of SRC in the nucleus [47,48]. A previous study showed that KANK2 inhibits caspase-independent apoptosis by preventing AIF from being released from mitochondria [26]. Meanwhile, KANK2 can suppress myocardial injury caused by acute pancreatitis through inhibiting the inflammatory response by deactivating p65 [49]. Although the roles of KANK2 have been extensively described, the interaction with HSP70 to protect lung injury from sepsis has not been reported. Our results revealed for the first time that binding of HSP70 with KANK2 regulated cell apoptosis, indicating that KANK2 is a critical protein for HSP70 protection.

As is well known, apoptosis can be induced by death-receptor-mediated and mitochondrion-mediated pathways. AIF is a classic mitochondrial protein that induces apoptosis in a caspase-independent way. Under normal circumstances, AIF is secluded behind the outer mitochondrial membrane. However, upon apoptosis, the permeability of the outer mitochondrial membrane (MOMP) is increased and AIF translocates to the cytosol and the nucleus, resulting in peripheral chromatin condensation and large-scale DNA fragmentation. In this study, we found that the interaction of HSP70 with KANK2 modulated translocation and release of AIF and impeded apoptosis. The anti-apoptotic function of HSP70 occurs through blocking the mitochondria to nucleus translocation of AIF. In gastric epithelial cells and growth retardation model triggered by *H. pylori*, downregulation of intracellular HSP70 led to an increase of AIF and cytosolic cytochrome C that contributes to the activation of apoptosis [50]. Furthermore, one research study conducted by Choudhury showed that there was also a significant increase in the nuclear accumulation of AIF in HSP70 KO mice compared with WT mice during ischemia/reperfusion [51]. Similar results were observed in HSP70 overexpression protecting neonatal hypoxic/ischemic brain as well [52]. In our study, using knockdown of KANK2 in overexpressed HSP70 A549 cells, we confirmed that the HSP70 inhibited AIF translocation and apoptosis through interacting with KANK2. Together, deletion of HSP70 increases AIF release into the cytoplasm on the mitochondrial apoptotic pathway, thereby exacerbating lung injury after CLP. HSP70 is vital to protect against apoptosis and lung injury associated with sepsis. These data are particularly important, as the significance of HSP70 has never been previously investigated in AIF-related apoptosis in the septic ALI model. This exploration provides a theoretical basis for the clinical application of HSP70 in sepsis treatment. In the future, we will utilize HSP70 truncations to figure out the region binding to KANK2 and synthesize effective peptides for treating septic lung injury.

## 5. Conclusions

In conclusion, we have demonstrated that HSP70 interacted with KANK2 to reduce apoptotic cell and that application of exogenous HSP70 is a novel potential therapeutic approach for lung injury secondary to sepsis by impairment of apoptotic cellular pathway and amelioration of pulmonary dysfunction secondary to CLP in mice.

## Data Availability

The datasets used and/or analyzed in the current study are available from the corresponding author upon reasonable request.

## Abbreviations

ALI	acute lung injury
HSP	heat shock protein
CLP	cecal ligation and puncture
OE	overexpress
KO	knockout
IP	immunoprecipitation
IB	immunoblotting
WB	Western blot
IF	immunofluorescence
KANK2	KN motif and ankyrin repeat domains 2
SRC	steroid receptor coactivator
SIP	SRC-interacting protein
AIF	apoptosis-inducing factor
TNF-α	tumor necrosis factor-α
IL-6	interleukin-6
ELISA	enzyme-linked immunosorbent assay
PCR	polymerase chain reaction
H&E	hematoxylin and eosin
si-RNA	small interfering RNA
IHC	immunohistochemistry
TUNEL	terminal deoxynucleotidyl transferase dUTP nick end labelling
h	hour
MSS	murine sepsis score
